# 'Tiny numbers' are actually tiny: Evidence from gestures in the TV News Archive

**DOI:** 10.1371/journal.pone.0242142

**Published:** 2020-11-17

**Authors:** Greg Woodin, Bodo Winter, Marcus Perlman, Jeannette Littlemore, Teenie Matlock

**Affiliations:** 1 English Language & Linguistics, University of Birmingham, Birmingham, United Kingdom; 2 Cognitive and Information Sciences, University of California, Merced, CA, United States of America; The Hong Kong Polytechnic University, HONG KONG

## Abstract

We report a large-scale, quantitative investigation of manual gestures that speakers perform when speaking metaphorically about numerical quantities. We used the TV News Archive–an online database of over 2 million English language news broadcasts–to examine 681 videos in which 584 speakers used the phrase 'tiny number', 'small number', 'large number', or 'huge number', which metaphorically frame numerical quantity in terms of physical size. We found that the gestures speakers used reflect a number of different strategies to express the metaphoric size of quantities. When referring to greater versus lesser quantities, speakers were far more likely to gesture (1) with an open versus closed hand configuration, (2) with an outward versus inward movement, and (3) with a wider distance between the gesturing hands. These patterns were often more pronounced for the phrases containing more extreme adjectives ('tiny/huge number'). However, we did not find that speakers performed two-handed versus one-handed gestures. Nor did we find that speakers performed right-handed versus left-handed gestures, when referring to greater versus lesser quantities. Overall, this work supports the claim that metaphoric thought is involved in the production of verbal metaphors that describe numerical magnitudes. It demonstrates that size-based numerical associations observed in previous lab experiments are active in real-life communication outside the lab.

## 1. Introduction

English speakers often talk about quantities in terms of physical size [1, 2] For instance, numbers of different magnitudes are typically described using size terms such as 'tiny', 'small', 'large', and 'huge', and changing quantities can be characterised as 'shrinking' or 'growing'. Using words or phrases from one domain (e.g., physical size) to describe another (e.g., numerical quantity) reflects what is referred to in cognitive linguistics as *conceptual metaphor* [3–5]. Conceptual metaphor theory views everyday linguistic expressions such as 'tiny number' as surface-level manifestations of mental schemas that people use to conceptualise numerical quantities.

Behavioural experiments support the idea that there is a deep mental connection between the conceptualisation of physical size and numerical quantity, as instantiated by linguistic metaphors. For instance, people are quicker to correctly judge which of two numbers is greater when the greater number is presented in larger typeface [6]. People are also quicker to correctly judge which of two dot displays contains more dots when the more numerous display covers more area [7]. Studies have linked these size-based spatial-numerical associations to manual actions [8]. For example, people are faster at initiating a precision grip (forefinger and thumb approaching one another, as if holding a small pellet) in response to lesser numbers, and initiating a power grip (firm grip involving the full hand, as if holding a pipe) in response to greater numbers [9]. Moreover, when people reach for blocks with numbers written on them, they spontaneously widen their grip aperture between index finger and thumb if the number is greater, regardless of the actual size of the blocks [10]. These studies show that thinking about greater numbers is mentally connected with actions used for interacting with larger objects, whereas thinking about lesser numbers is mentally connected with actions used for interacting with smaller objects.

Further evidence for the idea that linguistic metaphors such as 'tiny number' reflect a conceptualisation of quantity in terms of size comes from the gestures that speakers perform with their hands when talking about quantities. Winter and colleagues [11] discuss the gestures performed by a speaker on a TV news programme while making the following comment: 'There is a tiny number of people that are contributing a huge amount of money this election'. When saying 'tiny number', the speaker performed a gesture in which she drew her forefinger and thumb close together, as if holding the 'tiny number' between her fingers. When saying 'huge amount', she gestured outward from her body, with flat, open palms facing one another, as if representing the large physical size of the 'huge amount' with the space between her hands. Gestures such as these can be used as a window into the mind [12–14], and their potential to reveal metaphoric thought processes during language use has been shown for a number of abstract conceptual domains [15–18].

In this paper, we use gesture as a means of exploring mental number space. Much of the research on spatial-numerical association has focused on axial representations [2, 19, 20], but here we focus on the gestures that occur with linguistic expressions of size, specifically the metaphoric phrases 'tiny number', 'small number', 'large number', and 'huge number'. With gestures being a flexible way of expressing mental content, there is an array of possible strategies that speakers can use to express relative differences in size [21–24]. The experimental literature showing that precision grips are associated with small quantities [8–10] leads us to predict that speakers will use precision grips more often when talking about relatively smaller quantities. In addition, the fact that larger visually presented areas are associated with relatively larger quantities [6, 7] suggests that, when speakers demarcate a space between their hands, the distance between their hands should be wider when talking about greater quantities. Moreover, we may find that speakers will move their hands away from each other when talking about greater quantities, and toward each other when talking about smaller quantities.

The number of hands the speaker uses to gesture may also be associated with metaphoric size. For instance, while the distance between the thumb and index finger of a single hand can be used to depict smaller quantities, both hands may be needed to designate a wider space for representing greater quantities. In parallel to the use of precision-grip gestures–as if manipulating a small object–to represent lesser quantities, if greater quantities are conceptualised as physically larger, gestures may reflect two-handed manual actions associated with interacting with large physical objects. The use of both hands to represent greater numerical quantities may be comparable to the phenomenon of articulatory plurality in signed languages. Börstell and colleagues [25] showed in three historically unrelated signed languages (American Sign Language, Israeli Sign Language, Swedish Sign Language) that signs for plural concepts are more likely to be two-handed than one-handed. Here, we explore whether a similar effect can be observed for the manual gestures English speakers use when talking about numerical quantities, specifically, whether speakers are more likely to use two-handed gestures when talking about relatively greater magnitudes.

When speakers gesture with only one hand, the hand they opt to use may also reflect the way they conceptualise quantities of different magnitudes. There is evidence that English speakers think of numbers in terms of a mental number line, with lesser numbers being associated with leftward space and greater numbers with rightward space (e.g., the SNARC effect) [19, 20]. In line with this evidence, Daar and Praat [26] found that, when participants are given a free choice about whether to respond to numbers with their left or right hand, they are more likely to use their right hand in response to greater numbers. Furthermore, in a similar task involving the manual selection of number blocks, participants preferentially selected lesser numbers using their left hand [27]. Based on these results, speaker may be more likely to gesture with their left hand when talking about lesser quantities, and with their right hand when talking about greater quantities.

In addition to their binary magnitude, the four size-based metaphoric descriptions we investigate differ with respect to their position on the scalar dimension of size. Specifically, 'tiny number' and 'huge number' are more extreme than 'small number' and 'large number'. Thus, we were also able to examine whether the patterns of gesturing (e.g., open hand configurations for greater quantities) are more pronounced with the extreme phrases.

Our investigation of size gestures used to represent numerical quantities is important for several reasons. First, in contrast to experiments on numerical cognition, which typically require participants to respond to stimuli under highly constrained, laboratory-controlled conditions, gesture allows us to test the association between physical size and numerical quantity in a more ecologically valid medium: verbal communication. Gesture is a highly flexible modality of expression, especially in comparison to lab experiments, where speakers are typically forced to respond by pressing fixed response keys [2, 28]. During verbal communication, there is no requirement that speakers gesture at all, and the form and movement of these gestures is limited only by the mechanical constraints of the human body. Thus, the study of co-speech gestures can elucidate whether mental associations between space and number are evident in a less constrained and more naturalistic setting.

A second motivation behind our study is methodological. We demonstrate the value of a procedure for studying a large number of gestures produced in relation to specific verbal expressions. Gesture research is time-consuming and finding naturally-occurring gestures that evidence a specific phenomenon is difficult. Many gesture researchers annotate multimodal discourse for features of interest, scanning primary [29] or secondary [30] data for gestures relevant to their research topic. Even in studies where conversation between participants is elicited on a specific topic [31], unavoidably there are parts of the conversation that are irrelevant to the study's aims. In this study, we were able to examine hundreds of gestures that speakers produced when using specific verbal expressions (e.g., 'tiny number') by using the TV News Archive (https://archive.org/details/tv), a huge online database of television news shows, as well as public lectures and governmental programming. The archive, searchable by closed-caption transcripts, allowed us to automatically identify clips from news broadcasts featuring speakers–typically politicians, pundits, newscasters, and authors–using size-based metaphors to refer to quantity. All our data are therefore immediately relevant to our research questions, facilitating a bottom-up approach that is especially suited to the analysis of gestures occurring with particular linguistic expressions.

Another methodological advantage of using the TV News Archive as a data source is that it allows the collection of a large sample of videos and speakers. In combination, the number of videos (*N* = 681) and unique speakers (*N* = 584) in our dataset exceeds most previous gesture studies [18, 32, 33]. The quantitative focus of our study stands in contrast to observational gesture research, which has tended to focus on detailed qualitative analysis of particular examples [15, 28]. With this paper, we contribute to recent advances in large-scale, quantitative gesture research, particularly a recent study in this journal [34], which used the UCLA Red Hen Lab corpus to study hundreds of gestures related to time expressions [35]. In comparison to the Red Hen corpus, an advantage of the TV News Archive is that all its videos are immediately accessible to researchers, making our coding decisions fully transparent and reproducible [36]. Therefore, all videos used in this study and future studies conducted with the TV News Archive can be viewed and re-analysed by other scientists, without requiring registration or payment.

## 2. Methodology

### 2.1. The dataset

We began by downloading a list of URLs for 3200 videos selected at random from the TV News Archive that contained the phrases 'tiny number’, ‘small number’, ‘large number', and 'huge number' (800 videos per phrase), including plural phrases (e.g., 'huge numbers') and phrases interrupted by fillers (e.g., 'tiny erm number'). To do this, we used the statistical programming language R, version 3.5.1 [37], in the integrated development environment RStudio, version 1.1.456 [38], with the packages 'tidyverse', version 1.2.1 [47], 'rvest', version 0.3.3 [39], 'XML', version 3.98–1.20 [40], and 'jsonlite' [41]. The end result of this extraction was a spreadsheet with a list of video URLs that could be used to access the corresponding video in the TV News Archive. The scripts used to extract video URLs from the TV News Archive and the spreadsheet containing the URLs are publicly available at https://osf.io/dncjg/.

### 2.2. Video exclusion

Many videos did not lend themselves to informative gesture analysis. Videos were excluded from the final analyses for the following reasons:

- The video did not play or had no sound.
- The video was a duplicate copy of another video in the dataset.
- The speaker did not use the relevant phrase. These videos included false positives produced by the TV News Archive’s search engine and closed captioning system, inflections (e.g., 'smaller number'), videos where the words in the target phrase occurred in separate clauses (e.g., 'number one: voter turnout was small; number two: the election was rigged') or not as part of its own noun phrase (e.g., 'how large numbers will be'), and videos where the target phrase was interrupted by a non-filler lexical item (e.g., 'large negative numbers').
- The target phrase was preceded by another size adjective (e.g., 'small tiny numbers').
- The target phrase was negated (e.g., 'it is not a tiny number').
- There was not a sufficiently clear view of the speaker's hands to determine whether or not they gestured, or, if the gesture was visible, what hand configuration they used.
- The audio and video were desynchronized to the extent that it was difficult to determine whether the speaker's gesture co-occurred with their use of the target phrase.
- The speaker spoke a language other than English that was translated into English via voiceover.
- It was not physically practical for the speaker to gesture or change their hand configuration (e.g., they were holding a large object such as a clipboard or microphone) or their hands were engaged in some other task (e.g., shuffling papers).

After these exclusions were made, a total of 681 videos including 584 unique speakers underwent statistical analyses. Only 52 speakers appeared in more than one video, which amounted to 97 videos in total (14.2% of the whole dataset). 36 repeat speakers appeared twice, and 8 appeared three times. Table 1 shows the number of appearances for speakers that appeared in our dataset more than three times. The contribution of multiple data points by repeat speakers was factored into the design of our statistical models (see §2.5).

**Table 1:** Speakers that appeared in dataset more than three times and the number of times they appeared.

Speaker	Appearances
Jim Cramer	11
Donald Trump	8
Barack Obama	7
Bernie Sanders	6
Lawrence Lessig	6
Richard Wolff	6
Peter Lavelle	5
Joan Cashin	4

In the final dataset, 'tiny number' contributed 167 videos and 146 unique speakers, 'small number' contributed 170 videos and 157 unique speakers, 'large number' contributed 151 videos and 144 unique speakers, and 'huge number' contributed 193 videos and 179 unique speakers. Speakers referred to a wide range of actual quantities using these expressions, including 'millions', 'two tenths of one percent', 'one hundred', 'forty percent', and so on.

### 2.3. Manual gesture annotation

The first author manually coded the data, first by indicating whether or not the speaker gestured (subsequently referred to as Gesture Co-occurrence). To be counted as a gesture, we used the criterion that the speaker must produce a seemingly communicative movement that occurred at least partly in time with their use of the target phrase. While conservative, the criterion of temporal co-occurrence avoided the problem of subjectivity that arises when a gesture occurs in close proximity to a verbal expression that seems semantically related but whose semantic relation cannot be determined objectively. Instances where the speaker's hands were returning to rest position, or where the target phrase was uttered during the post-stroke hold of a gesture, with no additional movement, were not counted as gestures.

**Fig 1:**
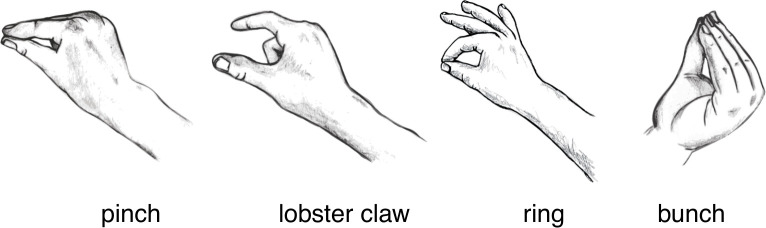
Illustrations of four different closed handshapes. The left three gestures involve precision grips (index finger and thumb approaching or touching each other). In what we call a ‘pinch’, the index finger and thumb are touching each other, or are held apart with a very narrow gap. In ‘lobster claw’ gestures, the index finger and thumb are held visibly apart. The ‘ring’ gesture is similar to the pinch, but with the middle, ring, and pinkie finger extended. For the ‘bunch’, speakers bring all fingers together, as if scraping together a heap of rice on a table.

The first author then determined the features of gestures using the following categories: Hand Configuration, Palm Orientation, Closed Handshape, Number of Hands, Hand Choice, Hand Distance, and Horizontal Movement. The first category, Hand Configuration, coded for whether the speaker gestured with an open or closed hand configuration. Open hand configurations involved the fingers being extended, such as when performing a palm-up open-hand gesture [42–44]. Open-hand gestures were further coded for Palm Orientation–whether the speaker's palms were facing predominantly upward, downward, inward (facing one another, toward the midline of the speaker's body), forward (away from the speaker), or backward (toward the speaker). The coding decisions for Palm Orientation were not connected to any specific hypothesis and so were not used in any inferential tests, but we report the overall results for Palm Orientation to provide a comprehensive description of the data. Closed hand configurations included gestures where the speaker draws the fingers on a single hand close together. These close-hand gestures were also coded for their specific Closed Handshape. As shown in Fig 1, these handshapes included three variants of precision grip-type gestures: 'pinch' gestures (with the index finger and thumb touching or approaching each other), 'lobster claw' gestures (with the index finger and thumb held further apart), and 'ring'-type gestures (with the forefinger and thumb touching and the middle, ring, and pinkie fingers extended, similar to the ‘OK’ emblem) [42]. Closed handshapes also included 'bunch' gestures (what Kendon calls a 'grappolo' [42], which involves all fingers being held together), clenched fists, and pointing gestures.

While coding the data, it became apparent that a small but significant subset of gestures were more curved inward and tense than open-hand gestures but were not sufficiently closed to constitute closed-hand gestures. We coded these curved gestures as a separate, in-between category. Because they were an intermediate case, we decided to exclude them from the statistical model that tested Hand Configuration, but we still report the number of occurrences of curved gestures across the four phrases in our descriptive analyses.

We also coded for several other properties of the gestures. Number of Hands coded for whether gestures were performed with one hand or two hands. For one-handed gestures, Hand Choice coded for whether the gesture was performed with the left hand or right hand. For two-handed gestures, Hand Distance coded the distance between the speaker's hands using the following categories: 1) narrow: the distance between the speaker's hands was less than the width of their head, 2) medium-width: the distance between the speaker's hands was equal to or more than the width of their head but less than the width of their torso, and 3) wide: the distance between the speaker's hands was equal to or more than the width of their torso. We used this categorical coding distance (using the speaker's body as a frame of reference) due to the fact that the camera position in the TV News Archive is not constant, making it impossible to measure gesture in terms of a more continuous measurement, such as the number of pixels between both hands. Finally, for two-handed gestures, Horizontal Movement coded for whether the speaker gestured with an inward movement (hands moving toward one another), or with an outward movement (hands moving away from one another).

### 2.4. Inter-rater reliability

A second coder (the third author of this paper) independently coded a subset of the videos to test for inter-rater reliability. First, 156 videos (22.9% of dataset) were used as a training set. During the training stage, disagreements between Coder 1 and Coder 2 were discussed and the coding scheme was updated (see OSF repository for coding scheme: https://osf.io/dncjg/). Following the training stage, 176 videos (25.8% of dataset) were coded by Coder 2. These 176 videos were coded for Gesture Co-occurrence, Hand Configuration, Palm Orientation, Closed Handshape, Number of Hands, Hand Choice, Hand Distance, and Horizontal Movement. We then calculated the inter-rater reliability (IRR) of coding decisions made by Coder 1 and Coder 2 with Cohen's kappa [45, 46] using the R package 'irr', version 0.84.1 [55]. The IRR results are shown in Table 2. Note that if a category was assigned a certain code for a particular video (e.g., Gesture Co-occurrence coded as 'no'), or if the coders could not agree on a code for this category (e.g., Coder 1 believed there was a gesture but Coder 2 did not), other categories were not coded (e.g., Hand Configuration). Because of this, the total number of videos coded for all columns to the right of Gesture Co-occurrence was fewer than 176.

**Table 2:** Inter-rater reliability between Coder 1 and Coder 2 of coding decisions relating to our hypotheses.

	Gesture Co-occurrence	Hand Configuration	Palm Orientation	Closed Handshape	Number of Hands	Hand Choice	Hand Distance	Horizontal Movement
**No. of videos**	176	127	88	31	129	68	53	54
**Agreement**	94.8%	96.1%	76.1%	67.7%	95.3%	95.6%	77.4%	87%
**Cohen’s kappa**	0.855	0.94	0.649	0.547	0.906	0.912	0.737	0.799
**Level of agreement**	Almost perfect	Almost perfect	Substantial	Moderate	Almost perfect	Almost perfect	Substantial	Substantial

We performed a weighted test for Hand Configuration to account for the fact that a disagreement over whether a hand is open or closed is a greater difference than a disagreement over whether a hand is curved or closed, or whether a hand is curved or open (curved gestures were in-between closed and open hand configurations). Similarly, we performed a weighted test for Hand Distance to account for the fact that a disagreement over whether a gesture is narrow or wide is a greater difference than a disagreement over whether a gesture is medium-width or narrow, or whether a gesture is medium-width or wide. We performed unweighted tests for the other categories because these coding decisions were not ordered.

For all columns except Closed Handshape, the IRR between Coder 1 and 2 was at least 'substantial', with 'almost perfect' agreement for Gesture Co-occurrence, Hand Configuration, Number of Hands, and Hand Choice. Given the lower coding reliability for Closed Handshape, we still report the descriptive statistics, but we do not perform any inferential tests.

Using a sample of videos from the current study and another investigation in progress comprising 69 videos in total, we addressed the concern that being able to hear the phrase used by the speaker may have affected gesture annotations. For example, if the coder knows that the phrase is 'huge number', this may bias them to analyse a hand configuration as open, rather than closed. To address this concern, we performed a round of blind coding. Blind coding was performed by a third coder (Samantha Ford) who viewed muted videos that were cut to the target phrase only, with the faces of speakers obscured to prevent lip reading. This round of coding focused on Gesture Co-occurrence and Hand Configuration, using a simplified version of the coding scheme that categorised gestures as either closed-hand or open-hand but not curved, as curved gestures were not included in our statistical models. For Gesture Co-occurrence, the IRR agreement was 92.8% (Cohen's *κ* = 0.666), indicating substantial agreement. For Hand Configuration, the IRR agreement was 94.6% (Cohen's *κ* = 0.838), indicating almost perfect agreement. Our main results are reported using the codes supplied by Coder 1.

### 2.5. Statistical analyses

All data were analysed with statistical programming software R, version 3.5.1 [37], in the integrated development environment RStudio, version 1.1.456 [38]. The package 'tidyverse', version 1.2.1 [47] was used for data processing and visualisation; 'ggmcmc' [48] and 'scales', version 1.0.0 [49] were used for data visualisation; 'brms' [50, 51] was used for Bayesian multilevel models; and 'irr', version 0.84.1 [52] was used to calculate inter-rater reliability. All data and analysis code are publicly available at https://osf.io/dncjg/. Statistical analyses were conducted to test the predictions summarised in Table 3.

**Table 3:** Summary of predictions for gestures we expected to observe alongside expressions referring to lesser and greater quantities; size of quantities increases from 'tiny number' to 'small number' to 'large number' to 'huge number'.

Predictions		
	Smaller quantities	Greater quantities
**Hand Configuration**	closed	open
**Hand Distance**	smaller distance between hands	larger distance between hands
**Horizontal Movement**	inward movement	outward movement
**Number of Hands**	one	two
**Hand Choice**	left	right

We used Bayesian logistic regression to test the hypotheses with categorical and binary dependent variables: Hand Configuration (closed versus open; curved gestures were not included in this model), Horizontal Movement (inward versus outward), Number of Hands (one-handed versus two-handed), and Hand Choice (left hand versus right hand). We used Bayesian ordinal regression to test the Hand Distance hypothesis, because this dependent variable had three ordered levels (narrow versus medium versus wide).

In all models, the sole predictor was Phrase ('tiny number', 'small number', 'large number', 'huge number'). This predictor was Helmert coded. Helmert coding compares each level of a categorical predictor variable to the mean of subsequent levels of that variable, allowing us to make the following comparisons: (1) 'small number' versus 'tiny number', (2) 'large number' versus 'small number' and 'tiny number', and (3) 'huge number' versus 'large number', 'small number', and 'tiny number'. This coding scheme allowed us to observe whether certain types of gestures (e.g., open-hand) became more frequent as the phrases referred to increasingly large quantities.

Random intercepts were included in all models to account for by-speaker variation in gestures. We did not include random slopes because most speakers did not appear in our dataset more than once, so they did not have repeated measures for the main predictors of interest (i.e., the predictors were almost exclusively between speakers, rather than within speakers).

We used default priors from the package 'brms' [50, 51] for intercept and standard deviation. We set weakly informative priors on fixed slopes (normal distribution centred at 0 with a standard deviation of 1) and random slopes (half-normal distribution centred at 0 with a standard deviation of 1). As our inference criterion, we observed whether the 95% credible intervals of the posterior distributions for each predictor included zero. Credible intervals that did not contain zero were interpreted as providing strong evidence for the effect of each predictor on the dependent variable. We also report the probability of each effect being above zero.

## 3. Results

**Fig 2:**
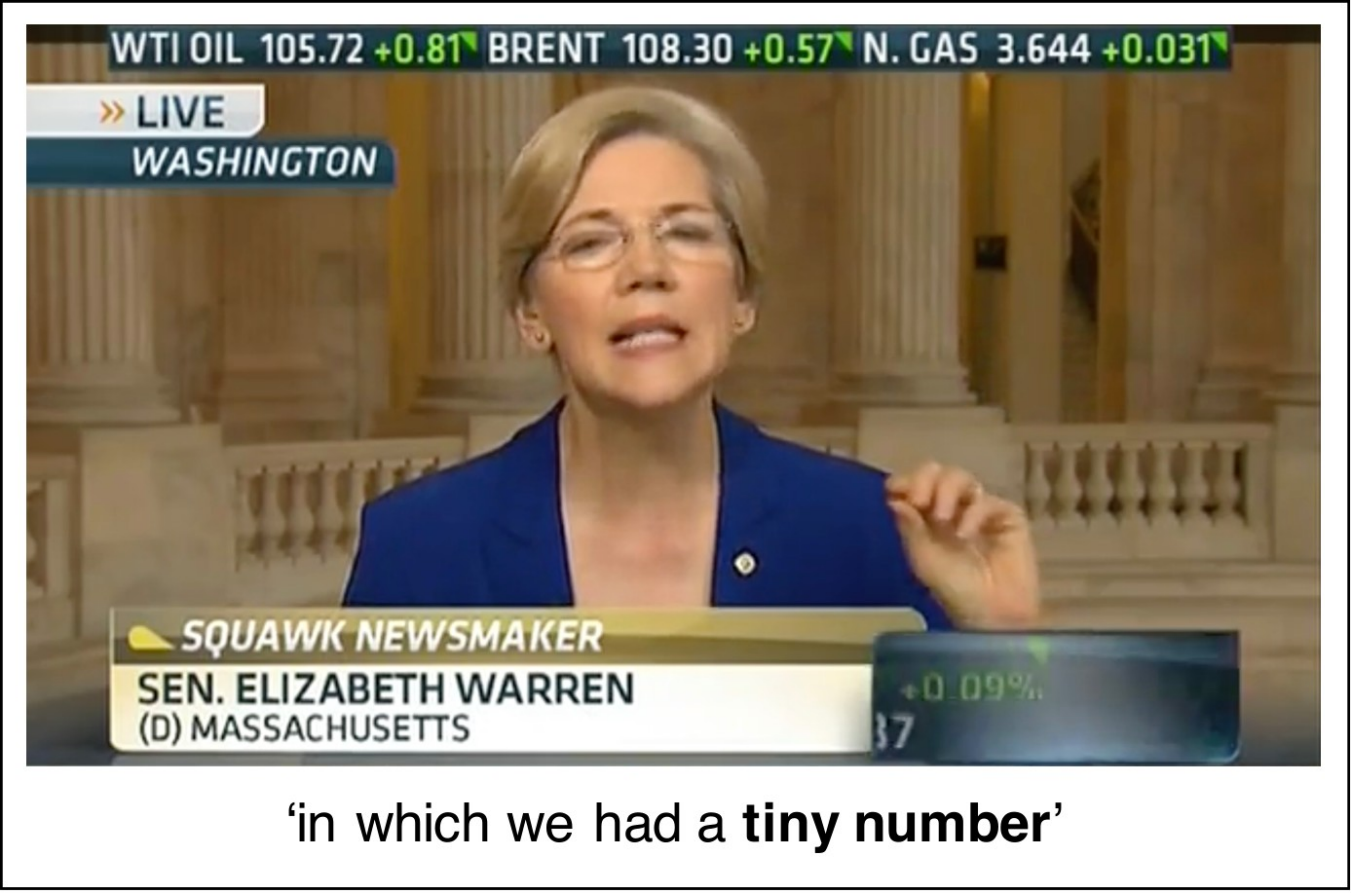
U.S. Senator Elizabeth Warren performs a pinch gesture with a closed hand configuration while using the target phrase 'tiny number', republished from CNBC (NBC Universal) under a CC BY license, with permission from CNBC (NBC Universal), original copyright 2013.

**Fig 3:**
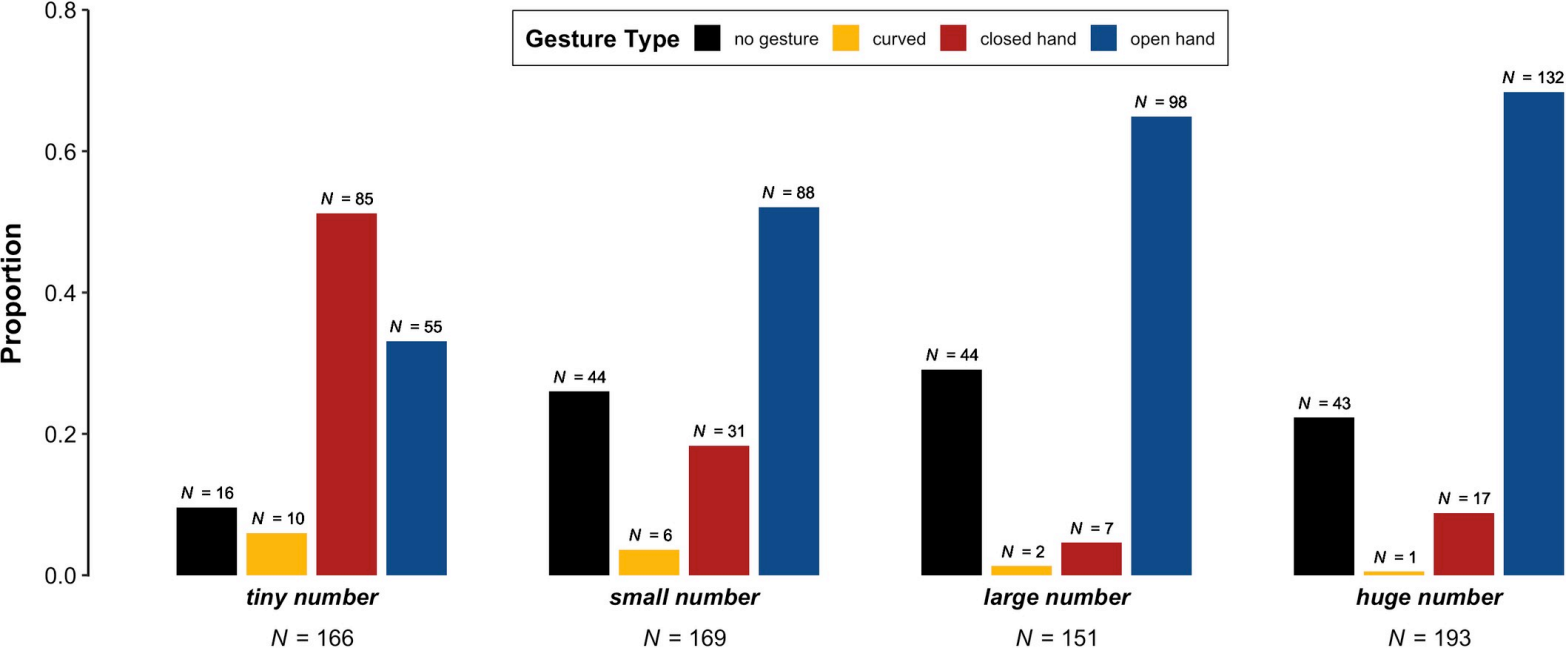
Proportion of videos with the phrases 'tiny number', 'small number', 'large number', and 'huge number' that occurred without a gesture or with a gesture in a curved, closed, or open hand configuration.

Fig 2 shows an example of a speaker performing a gesture in a video from our dataset. Fig 3 shows the proportions of videos for each phrase in which speakers did or did not gesture. The proportions displayed in the figure are based on all videos, excluding two videos in which the speaker's hands were in different configurations (left hand open, right hand closed).

Overall, more videos contained gestures (78.4%, *N* = 534) than did not contain gestures (21.6%, *N* = 147), including the two videos excluded from the figure. The expression 'tiny number' had the highest rate of gesture co-occurrence (90.4%, *N* = 151), with comparatively few videos not containing gestures (9.6%, *N* = 16). For the other three phrases, gesture co-occurrence was lower but still relatively high: of these three phrases, 'huge number' had the highest rate of gesture co-occurrence (gesture: 77.7%, *N* = 150; no gesture: 22.3%, *N* = 43), followed by 'small number' (gesture: 74.1%, *N* = 126; no gesture: 25.9%, *N* = 44), and then 'large number' (gesture: 70.9%, *N* = 107; no gesture: 29.1%, *N* = 44).

### 3.1. Hand configuration

We now look at whether speakers performed gestures with closed or open hand configurations. In the following analysis, we exclude two gestures where the speaker's hands were in different configurations (left hand open, right hand closed). The proportions reported in the following paragraph are based solely on closed-hand and open-hand gestures.

As shown in Fig 3, 'tiny number' is the only phrase that was accompanied by closed-hand gestures in a majority of videos (60.7%, *N* = 85), with fewer open-hand gestures (39.3%, *N* = 55). In contrast, 'large number' was mostly accompanied by open-hand gestures (93.3%, *N* = 98), with comparatively few closed-hand gestures (6.7%, *N* = 7). For 'huge number', open-hand gestures were similarly dominant (88.6%, *N* = 132), with comparatively few closed-hand gestures (11.4%, *N* = 17). For 'small number', open-hand gestures still predominated (73.9%, *N* = 88), but closed-hand gestures were relatively more frequent (26.1%, *N* = 31) compared to the phrases referring to large quantities. In fact, there were more closed-hand gestures for 'small number' than for 'large number' and 'huge number' put together.

The posterior distributions of the Bayesian logistic regression model (see §2.3 for model specification) provide strong evidence for an effect of Phrase ('tiny number', 'small number', 'large number', 'huge number') on Hand Configuration (closed versus open). For all comparisons, credible intervals did not include zero. In addition, the percentage of posterior samples that were above zero for each comparison was exactly or near 100%. First, open-hand gestures were more frequent alongside 'small number' than 'tiny number' (Helmert-coded odds = 2.29 to 1, log odds = 0.83, Bayesian 95% credible interval = [0.51, 1.23], posterior samples > 0 = 100%). Second, open-hand gestures were more frequent alongside 'large number' than 'tiny number' and 'small number' (odds = 2.34 to 1, log odds = 0.85, 95% *CI* = [0.55, 1.20], post > 0 = 100%). Third, open-hand gestures were more frequent alongside 'huge number' than 'tiny number', 'small number', and 'large number' (odds = 1.3 to 1, log odds = 0.26, 95% *CI* = [0.11, 0.44], post > 0 = 99.9%). Thus, the Helmert coded predictor shows that there were progressively more open hand configurations for progressively greater quantities.

Other gestures not included in the above analysis were coded as 'curved', being midway between closed and open hand configurations. The following proportions are based solely on curved gestures. Most curved gestures appeared alongside the phrase 'tiny number' (52.6%, *N* = 10), followed by 'small number' (31.6%, *N* = 6), 'large number' (10.5%, *N* = 2), and then 'huge number' (5.3%, *N* = 1). Thus, although these gestures were not very frequent overall, they seemed to be more commonly associated with lesser quantities.

For closed-hand gestures, we annotated the specific type of Closed Handshape. The following proportions are based solely on closed-hand gestures. For the expressions referring to smaller quantities, pinch gestures were the most prevalent gesture type ('tiny number': 45.8%, *N* = 38; 'small number': 48.4%, *N* = 15), followed by lobster claw gestures ('tiny number': 24.1%, *N* = 20; 'small number': 29%, *N* = 9), and then bunch gestures ('tiny number': 14.5%, *N* = 12; 'small number': 19.4%, *N* = 6). Less prevalent or absent were ring-type gestures ('tiny number': 7.2%, *N* = 5; 'small number': 0%, *N* = 0), pointing gestures ('tiny number': 4.8%, *N* = 4; 'small number': 0%, *N* = 0), and clenched fist gestures ('tiny number': 3.6%, *N* = 3; 'small number': 3.2%, *N* = 1). The counts for 'large number' are low and so do not present any interpretable pattern (7 gestures across 5 categories). However, the counts for 'huge number' reveal that pointing gestures predominated (41.2%, *N* = 7), followed by clenched fist gestures (29.4%, *N* = 5). Gestures in the other categories were at similarly low levels (ring-type: *N* = 2; bunch: *N* = 1; lobster claw: *N* = 1; pinch: *N* = 1).

For open-hand gestures, we also coded for Palm Orientation. The following proportions are based solely on open-hand gestures, minus 9 videos we excluded for containing gestures where the speaker's palms were oriented differently from one another (e.g., left palm downward, right palm inward). For three of the four phrases, inward facing palms (facing each other, toward midline of speaker's body) were the most common orientation: 'tiny number' (59.3%, *N* = 32), 'small number' (55.8%, *N* = 48), and 'huge number' (51.9%, *N* = 67). For 'large number', inward facing palms were less common (40%, *N* = 38). The general dominance of inward facing palms makes sense given that this gesture type is a prototypical size gesture, where the distance between the palms represents the size of the referent [53]. Compared to inward facing palms, upward facing palms were less common across the four phrases, with 'large number' (20%, *N* = 19) having a slightly higher proportion of this palm orientation than 'huge number' (17.8%, *N* = 23), which had a higher proportion of upward facing palms than both 'tiny number' (14.8%, *N* = 8) and 'small number' (14%, *N* = 12). For the phrases that referred to greater quantities, backward facing palms were more common ('large number': 13.7%, *N* = 13; 'huge number': 10.1%, *N* = 13) than for the phrases that referred to lesser quantities ('tiny number': 5.6%, *N* = 3; 'small number': 4.7%, *N* = 4). Similarly, for the greater quantity phrases, downward facing palms were more common ('large number': 15.8%, *N* = 13; 'huge number': 15.5%, *N* = 20) than for the lesser quantity phrases ('tiny number': 11.1%, *N* = 6; 'small number': 11.6%, *N* = 10). Lastly, 'small number' had a higher proportion of frontward facing palms (14%, *N* = 12) than 'large number' (10.5%, *N* = 10) and 'tiny number' (9.3%, *N* = 5) with the lowest proportion of frontward facing palms accompanying 'huge number' (4.7%, *N* = 6).

### 3.2. Number of hands

**Fig 4:**
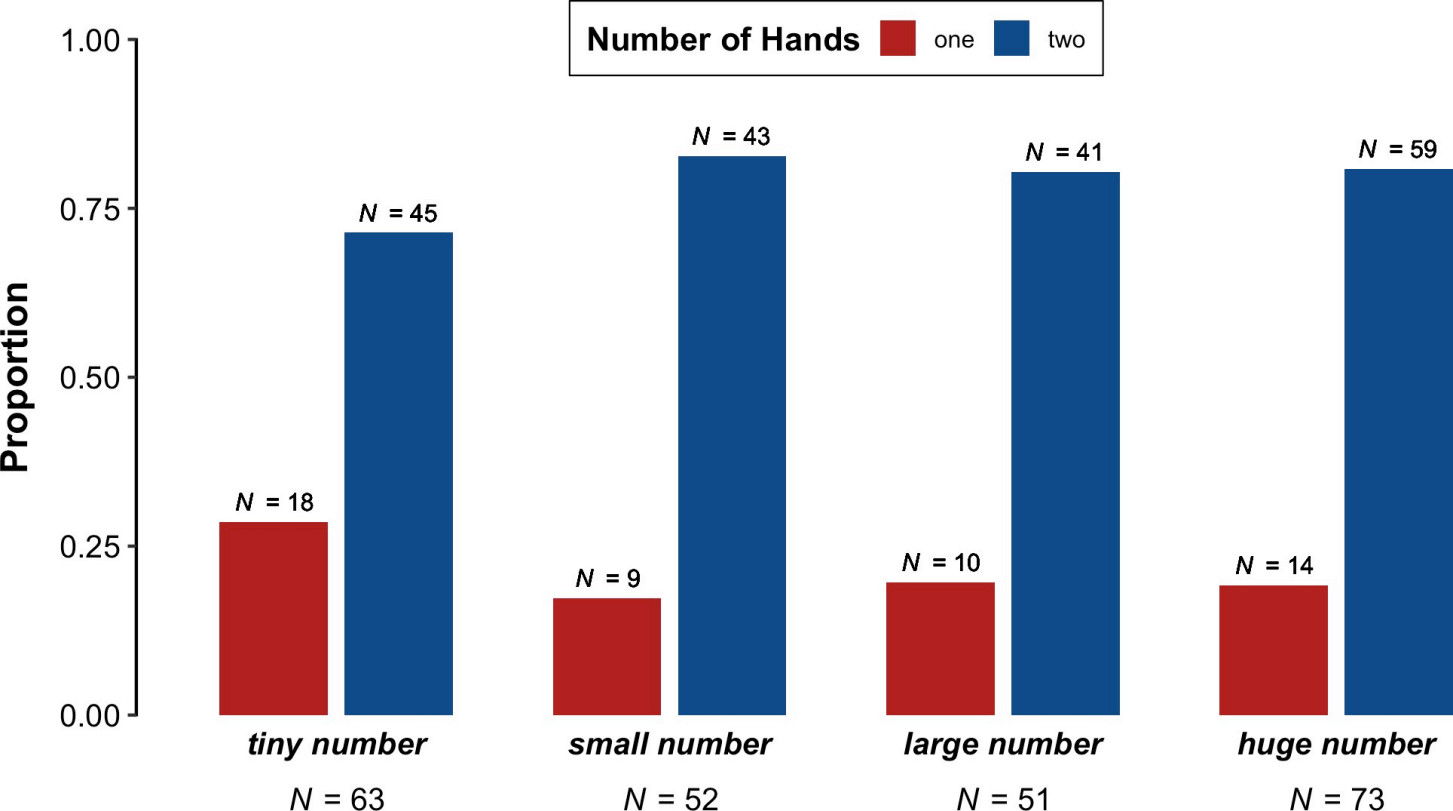
Proportion of videos in which both speakers' hands were visible and free to gesture per phrase ('tiny number', small number', 'large number', 'huge number') that contained one-handed versus two-handed gestures.

We now look at whether speakers were more likely to gesture with one hand or two hands when using the target expressions. The following proportions are based on all videos in which both speakers' hands were visible and free to gesture. Fig 4 shows the results from this analysis. Descriptively, 'tiny number' had the highest proportion of one-handed gestures (71.4%, 45 gestures), with fewer two-handed gestures (28.6%, 18 gestures). The proportions of one-handed and two-handed gestures were similar for 'small number' (one hand: 17.3%, 9 gestures; two hands: 80.4%, 43 gestures), 'large number' (one hand: 19.6%, 10 gestures; two hands: 80.4%, 41), and 'huge number' (one hand: 19.2%, 14 gestures; two hands: 80.8%, 59 gestures).

The posterior distributions of the Bayesian logistic regression model do not provide evidence for an effect of Phrase ('tiny number', 'small number', 'large number', 'huge number') on Number of Hands (one versus two). First, the model did not reveal a reliable difference between 'small number' and 'tiny number', with a credible interval that included zero (odds = 1.48 to 1, log odds = 0.39, 95% *CI* = [-0.11, 0.97], post > 0 = 93.7%). Similarly, the model did not indicate that there was a reliable difference between 'large number' and the phrases 'tiny number' and 'small number' (odds = 1.07 to 1, log odds = 0.07, 95% *CI* = [-0.24, 0.39], post > 0 = 65.7%). Finally, the model did not indicate that there was a reliable difference between 'huge number' compared to 'tiny number', 'small number', and 'large number' (odds = 1.03 to 1, log odds = 0.03, 95% *CI* = [-0.17, 0.24], post > 0 = 62.3%).

### 3.3. Hand distance

**Fig 5:**
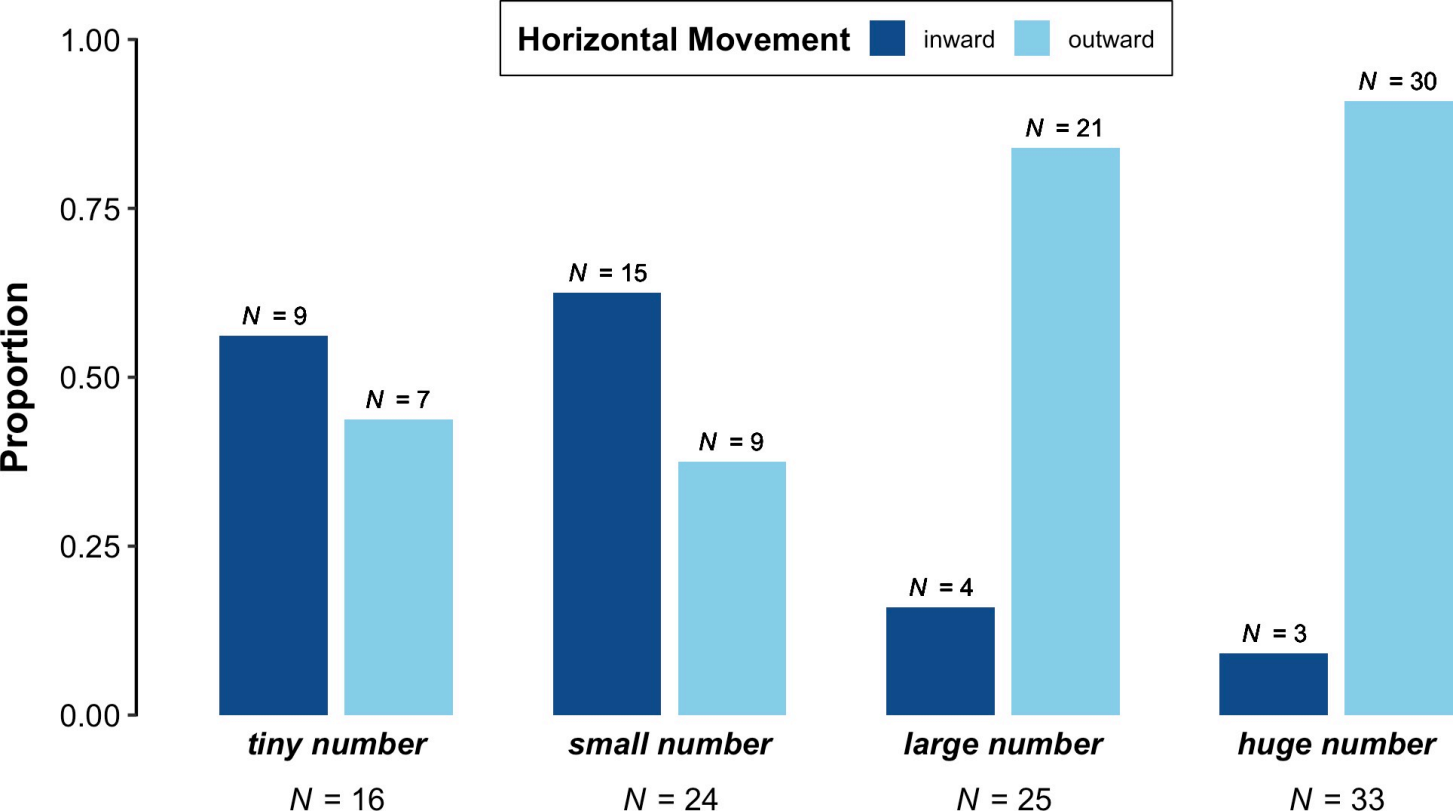
Proportion of two-handed gestures per phrase ('tiny number', small number', 'large number', 'huge number') that were inward moving versus outward moving.

For two-handed gestures, we now look at the distance between the speaker's hands at the end of the gesture stroke. The following proportions are based on all two-handed gestures, minus 6 gestures we excluded because it was not possible to determine the distance between the speaker's hands (e.g., the speaker's hands disappeared offscreen before the end of the gesture stroke). As shown in Fig 5, the phrases that referred to lesser quantities had a higher proportion of narrow gestures ('tiny number': 70.8%, 34 gestures; 'small number': 68.2%, 30 gestures) than 'large number' (41.5%, 17 gestures) and especially 'huge number' (30.8%, 20 gestures). In comparison, the lesser quantity expressions had a lower proportion of medium-width gestures ('small number': 22.7%, 10 gestures; 'tiny number': 18.8%, 9 gestures) compared to 'huge number' (29.2%, 19 gestures) and especially 'large number' (36.6%, 15 gestures). Similarly, the lesser quantity expressions had a lower proportion of wide gestures ('tiny number': 10.4%, 5 gestures; 'small number': 9.1%, 4 gestures) compared to 'large number' (22%, 9 gestures) and especially 'huge number', for which wide gestures was the most common hand distance (40%, 26 gestures).

The posterior distributions for the Bayesian ordinal regression model (outlined in §2.3) provide some evidence for an effect of Phrase ('tiny number', 'small number', 'large number', 'huge number') on Hand Distance (narrow versus medium versus wide). As a caveat, the model indicated that there was not a reliable difference between 'small number' and 'tiny number' (odds = 1.07 to 1, log odds = 0.07, 95% *CI* = [-0.51, 0.67], post > 0 = 59.6%). However, there was strong evidence that wider gestures were more frequent alongside 'large number' than alongside 'tiny number' and 'small number' (odds = 1.59 to 1, log odds = 0.5, 95% *CI* = [0.15, 0.85], post > 0 = 99.7%). There was also strong evidence that wider gestures were more frequent alongside 'huge number' than alongside 'tiny number', 'small number', and 'large number' (odds = 1.65 to 1, log odds = 0.5, 95% *CI* = [0.27, 0.82], post > 0 = 100%). In sum, there was a trend for speakers to gesture with a larger distance between their hands when using the phrases referring to greater quantities ('large number', 'huge number') than when using the phrases referring to lesser quantities ('tiny number', 'small number'). Speakers tended to perform gestures with a particularly large distance between the hands for 'huge number'.

### 3.4. Horizontal movement

We now examine whether speakers performed two-handed gestures with an inward movement (hands moving toward each other) or an outward movement (hands moving away from each other) when using the target expressions. The proportions reported in this section are based solely on two-handed gestures, minus 92 two-handed gestures in which the speaker's hands did not move in a horizontal direction, and 14 two-handed gestures where both hands moved horizontally in the same direction (see OSF repository for distribution of the excluded gestures across the four phrases: https://osf.io/dncjg/).

**Fig 6:**
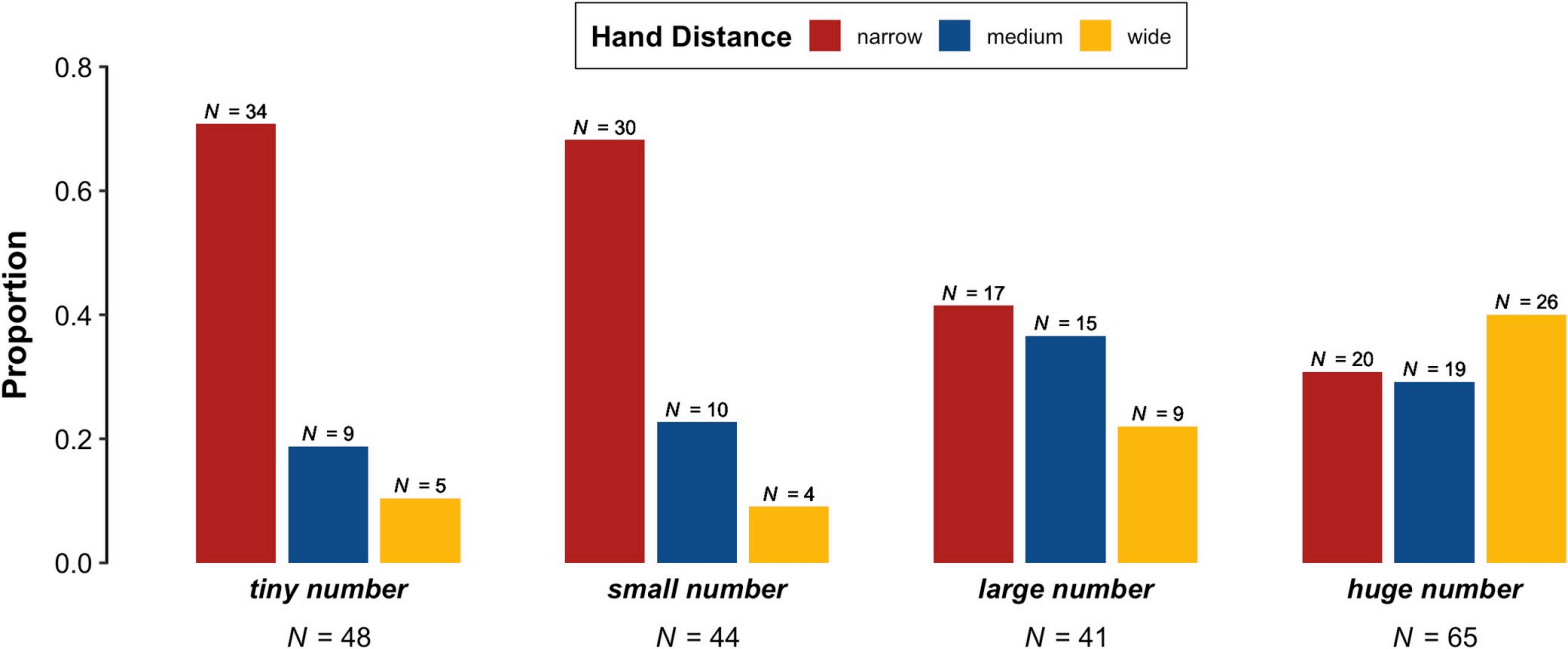
Proportion of two-handed gestures per phrase ('tiny number', small number', 'large number', 'huge number') in which the speaker's hands were separated by a narrow, medium, or wide distance.

As shown in Fig 6, 'tiny number' (inward: 56.2%, 9 gestures; outward: 43.8%, 7 gestures) and 'small number' (inward: 62.5%, 15 gestures; outward: 37.5%, 9 gestures) were accompanied mostly by inward-moving gestures. In contrast, 'large number' (inward: 16%, 4 gestures; outward: 84%, 21 gestures) and 'huge number' (inward: 9.1%, 3 gestures; outward: 90.9%, 30 gestures) were accompanied mostly by outward-moving gestures.

Overall, the posterior distributions of the Bayesian logistic regression model provide strong evidence for an effect of Phrase ('tiny number', 'small number', 'large number', 'huge number') on Horizontal Movement (inward versus outward). As with Hand Distance, 'tiny number' and 'small number' did not appear to be reliably different (odds = 0.83 to 1, log odds = -0.18, 95% *CI* = [-1.02, 0.61], post > 0 = 32.5%). However, the model indicated that outward-moving gestures were more frequent alongside 'large number' than 'tiny number' and 'small number' (odds = 2.36 to 1, log odds = 0.86, 95% *CI* = [0.34, 1.53], post > 0 = 100%). Furthermore, the model indicated that outward-moving gestures were more frequent alongside 'huge number' than 'tiny number', 'small number', and 'large number' (odds = 1.9 to 1, log odds = 0.64, 95% *CI* = [0.25, 1.16], post > 0 = 100%). The results of this model thus confirm the pattern depicted in Fig 6: the phrases referring to lesser quantities ('tiny number' and 'small number') were accompanied mostly by inward-moving gestures, whereas the phrases referring to greater quantities ('large number' and 'huge number') were accompanied mostly by outward-moving gestures. In addition, 'huge number' was accompanied by a higher proportion of outward-moving gestures than 'large number'.

### 3.5. Hand choice

**Fig 7:**
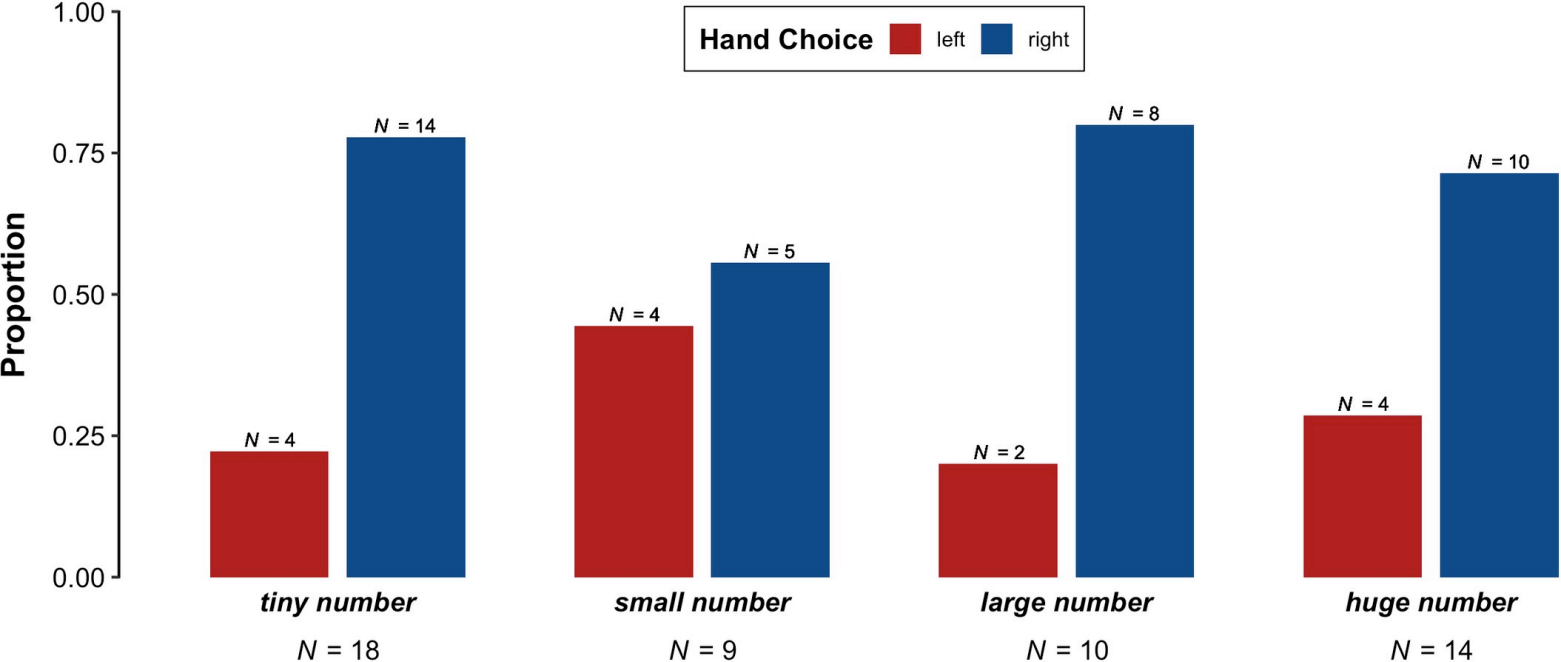
Proportion of videos in which both speakers' hands were visible and free to gesture per phrase ('tiny number', 'small number', 'large number', 'huge number') that contained left-handed versus right-handed gestures.

Finally, we investigate whether speakers gestured with their left hand or right hand when using the target expressions. The following proportions are based on all one-handed gestures in which the speaker's hands were both visible and free to gesture. Because there were relatively few of this type of gesture, the counts reported here are low, but there was a clear trend for right-handed gestures to predominate, overall. As shown in Fig 7, descriptively, 'large number' had the highest proportion of right-handed gestures (80%, *N* = 8) and the lowest proportion of left-handed gestures (20%, *N* = 2). Similarly, 'tiny number' had a high proportion of right-handed gestures (77.8%, *N* = 14) and a low proportion of left-handed gestures (22.2%, *N* = 4). For 'huge number', right-handed gestures were slightly less frequent but still predominated (71.4%, *N* = 10), while left-handed gestures were slightly more frequent but still low in frequency (28.6%, *N* = 4). Finally, 'small number' had the lowest proportion of right-handed gestures (55.6%, *N* = 5) and the highest proportion of left-handed gestures (44.4%, *N* = 4).

The posterior distributions of the Bayesian logistic regression model do not provide strong evidence for an effect of Phrase ('tiny number', 'small number', 'large number', 'huge number') on Hand Choice (left versus right). The credible intervals for each comparison included zero: between 'small number' and 'tiny number' (odds = 0.61 to 1, log odds = -0.49, 95% *CI* = [-1.49, 0.48], post > 0 = 15.7%), between 'large number' and the phrases 'tiny number' and 'small number' (odds = 0.98 to 1, log odds = 0.26, 95% *CI* = [-0.43, 1.01], post > 0 = 76.1%), and between 'huge number' and the phrases 'tiny number', 'small number', and 'large number' (odds = 1.03 to 1, log odds = -0.01, 95% *CI* = [-0.46, 0.47], post > 0 = 47.1%). Therefore, we do not find any support for the hypothesis that right-handed gestures more frequently accompany phrases referring to greater quantities.

## 4. Discussion

We used the TV News Archive to conduct a large-scale, quantitative investigation of the gestures that speakers produce when talking about numerical quantity. Specifically, we examined whether their gestures reflect the size-based verbal metaphors they use when referring to quantities of different magnitudes. Our search included four metaphoric expressions: ‘tiny number’, ‘small number’, ‘large number’, and ‘huge number’. This search returned 681 videos that met our inclusion criteria (speakers visible, hands free, etc.), with 534 gestures performed by 461 speakers coded in total.

Gesture co-occurrence rates ranged from 77.7% for 'large number' to 90.4% for 'tiny number', demonstrating that speakers were much more likely to gesture than not across the four phrases. Of the gestures that occurred, our results showed that speakers used a wide array of strategies for signalling relative differences in size via gesture: changing their hand configuration, changing the space spanned between their hands, and moving their hands inward or outward. These results indicate that the associations between precision grips and small quantities [8–10], and between larger visually presented areas and greater quantities [6, 7] observed in laboratory experiments, extend to communicative actions produced spontaneously during verbal communication.

The predominance of open-hand gestures for three of the four phrases in the dataset may indicate that open hand configurations are the gestural norm or default, perhaps because the hands may tend to be in an approximately open hand configuration while at rest, making it easier to perform an open-hand gesture than a closed-hand one. To override this default, a sufficiently strong association between a numerical quantity and closed-hand actions may be required [9, 10, 54], which appears to be triggered most reliably when speakers use the phrase 'tiny number', even more so than the less extreme expression 'small number'.

Of the precision grip-type gestures that occurred alongside the expressions referring to lesser quantities ('tiny number', 'small number'), pinches were most frequent, followed by lobster claw gestures. In contrast, bunch gestures occurred comparatively less frequently. Interestingly, the ring gesture (where the middle, ring, and pinkie finger are extended) was much less frequent than the pinch and the lobster claw, both of which involve curling in the middle, ring, and pinkie finger. These results are in line with Hassemer and Winter’s [21, 22] analysis of precision grips, according to which curling in all fingers other than the profiled index finger and thumb is crucial for the expression of size information, particularly for small referents. Another potential explanation is that the ring gesture is similar to the 'OK' emblem [42], and so it may be avoided because of its association with this highly conventionalised meaning, which is unrelated to size.

When speakers referred to greater quantities ('large number', 'huge number'), they also tended to gesture with a wider distance between their hands, and were more likely to gesture with an outward movement, than when they referred to lesser quantities ('tiny number', 'small number'). While we treated the distance between speakers' hands and their movement direction separately in our analysis, they are often two facets of the same gesture: speakers tended to move their hands apart to a wide distance when talking about greater quantities, and move their hands to a narrow distance when talking about lesser quantities. Specifically, 77.4% (*N* = 24) of inward-moving gestures were narrow, whereas 12.9% (*N* = 4) were medium-width, and 9.7% (*N* = 3) were wide. In contrast, just 21% (*N* = 13) of outward-moving gestures were narrow, 32.3% (*N* = 20) were medium-width, and 46.8% (*N* = 29) were wide.

We did not find any overall two-handed bias for greater quantities, as we expected based on the observation that two-handed actions are more compatible with larger physical quantities, as well as the sign language phenomenon of articulatory plurality [25]. We also did not find any evidence that speakers were more likely to produce right-handed gestures for greater quantities, which we predicted based on the experimental finding that quantities are mentally represented with lesser quantities on the left to and greater quantities on the right [19, 20], in addition to previous research demonstrating patterns in the hand chosen to respond to different numerical magnitudes [26, 27]. Our results suggest that these experimental findings may not apply to gestures produced spontaneously when speakers use size-based expressions. Instead, consistent with the verbal metaphors that speakers used, speakers appeared to be conceptualising the numbers primarily in terms of physical size, rather than the horizontal axis.

Our results support the core claim of Conceptual Metaphor Theory that verbal metaphors reflect mental schemas that people use to conceptualise different aspects of the world [3–5]. At least with respect to the numerical quantity metaphors in our dataset, the patterns we observed in speakers' gestures suggested that these metaphors are not mere figures of speech, but rather represent a deeper, size-based conceptualisation of numerical quantities. The foregrounding of verbal metaphors through gesture can thus be seen as an additional source of evidence that metaphors are conceptually 'active' during the production of an utterance [18–21]. This is especially important given that expressions such as 'large number' are highly conventionalised. Conventionalisation is framed as a serial killer of metaphoricity–as conventionalisation increases, the likelihood that speakers will recognise an expression's metaphoric properties is thought to decrease [55–58]. Despite conventionalisation, the gestures observed in our dataset suggest that the particular metaphors we investigated were conceptually active for many speakers.

Because speakers used explicitly size-based language, we cannot rule out the possibility that language itself is the primary driver of the gestures we observed, without there being any deeper conceptual mapping. According to Bouissac [59], a gesture co-occurring with a metaphoric expression may redundantly imitate speech, which would make the gesture *iconically* depictive of the concept expressed in speech, but not metaphoric. However, to the extent that size-based words such as 'tiny' and 'huge' are used to describe an abstract domain (i.e., numerical quantity) in expressions such as 'tiny number' and 'huge number', the whole phrase can be seen as metaphoric. In that case, the iconic gestures corresponding to these words would still occur within an overall abstract context and are metaphoric at least to this degree. Furthermore, while lexical priming of gestures is a possibility for the present dataset, other research has provided evidence for the existence of conceptual metaphors in language-independent tasks that do not use verbal metaphors as prompts [60–62]. Finally, gesture research has shown that speakers sometimes produce metaphoric gestures while not expressing these metaphors verbally [28, 63, 64]. These three points notwithstanding, future research with the TV News Archive can address this concern empirically by investigating the gestures that correlate with expressions that do not relate directly to size, such as 'more', 'less', or number words.

Taken together, our results show how cognitive associations previously observed in experiments extend to language use outside the laboratory–in a database of television news shows, public lectures, and governmental programming, featuring politicians, pundits, newscasters, and authors. This methodological contribution is important for several reasons. First, as experimental methods in research on spatial associations inherently constrain participants' behaviour and thus are subject to task demands. In contrast, gesture is a much freer form of expression. A further limitation of lab-based studies on spatial-numerical associations [20, 65] is that a small set of stimuli is typically explored–often for instance, the numbers 1 to 9. For the verbal metaphors investigated here, speakers referred to a wide range of numerical magnitudes; for instance, 'millions', 'two tenths of one percent', 'one hundred', 'forty percent', and so on. That we found congruent gestures across most of these different quantities shows that the large experimental body of spatial-numerical associations extends to vast numerical ranges spanning many orders of magnitude.

The TV News Archive also offers the advantage that it enables the collection of far more data, especially a higher number of speakers, than would usually be feasible in an experimental setting. Moreover, if time-consuming stages of the analysis procedure could be automated, such as the initial exclusion of unusable videos, still larger databases could be constructed (see [66]), and large portions of the analysis itself could be automated using the Python-operated OpenPose software [67, 68] (see [69, 70]). Finally, the data in the Archive are openly accessible, allowing completely reproducible analyses to be conducted. Therefore, we believe the TV News Archive to be a promising tool for gesture research, for use alongside the popular UCLA Red Hen Lab corpus [35].

However, while our data are arguably more ecologically valid than results obtained via experimentation, it is not clear to what extent gestures produced in televised contexts reflect natural communication. For instance, some politicians and newsreaders that appear in our dataset are likely to have received body language training [71], and may be engaged in non-standard communicative practices such as reading from a script or speaking to a camera, which may affect their gestures. We thus argue for the triangulation of results from different research paradigms when making broad claims about cognition based on gestures.

To conclude, in this study, we have provided large-scale, quantitative evidence to show how conceptual associations between size and numerical quantity cited in the research literature are commonly foregrounded through speakers’ gestures when they use different metaphors in their speech. In particular, greater quantities are often conceptualised as physically larger than lesser quantities, and speakers can represent this conceptualisation through the different kinds of gestures that they produce, using their hand configuration, the movement of their hands, and the distance between them. The gestural patterns we observed support the idea that verbal metaphors such as 'tiny number' are psychologically real: 'tiny numbers' are actually tiny, at least in the minds of speakers. These results show how lab-based research on spatial-numerical associations and other conceptual metaphors can benefit from investigating language and gesture, providing converging evidence for the same underlying conceptual mappings. More broadly, our analyses show how speakers’ gestures can provide a window into their internal mental processes, making these processes visible to the researcher. Thus, by applying quantitative methods to large samples of different speakers across different contexts, we can use gesture to observe trends in the way the human mind works.
